# Supplementary material for: An Endosomal NAADP-Sensitive Two-Pore Ca2+ Channel Regulates ER-Endosome Membrane Contact Sites to Control Growth Factor Signaling
Source: Cell Rep. 2017 Feb 14;18(7):1636–45. doi: 10.1016/j.celrep.2017.01.052 (PMC5318655; doi:10.1016/j.celrep.2017.01.052)
Supplement: Document S1. Figures S1–S4 and Table S1 [file mmc1.pdf]

Cell Reports, Volume *18*

Supplemental Information

An Endosomal NAADP-Sensitive Two-Pore Ca^{2+} Channel Regulates ER-Endosome Membrane Contact Sites to Control Growth Factor Signaling

Bethan S. Kilpatrick, Emily R. Eden, Leanne N. Hockey, Elizabeth Yates, Clare E. Futter, and Sandip Patel

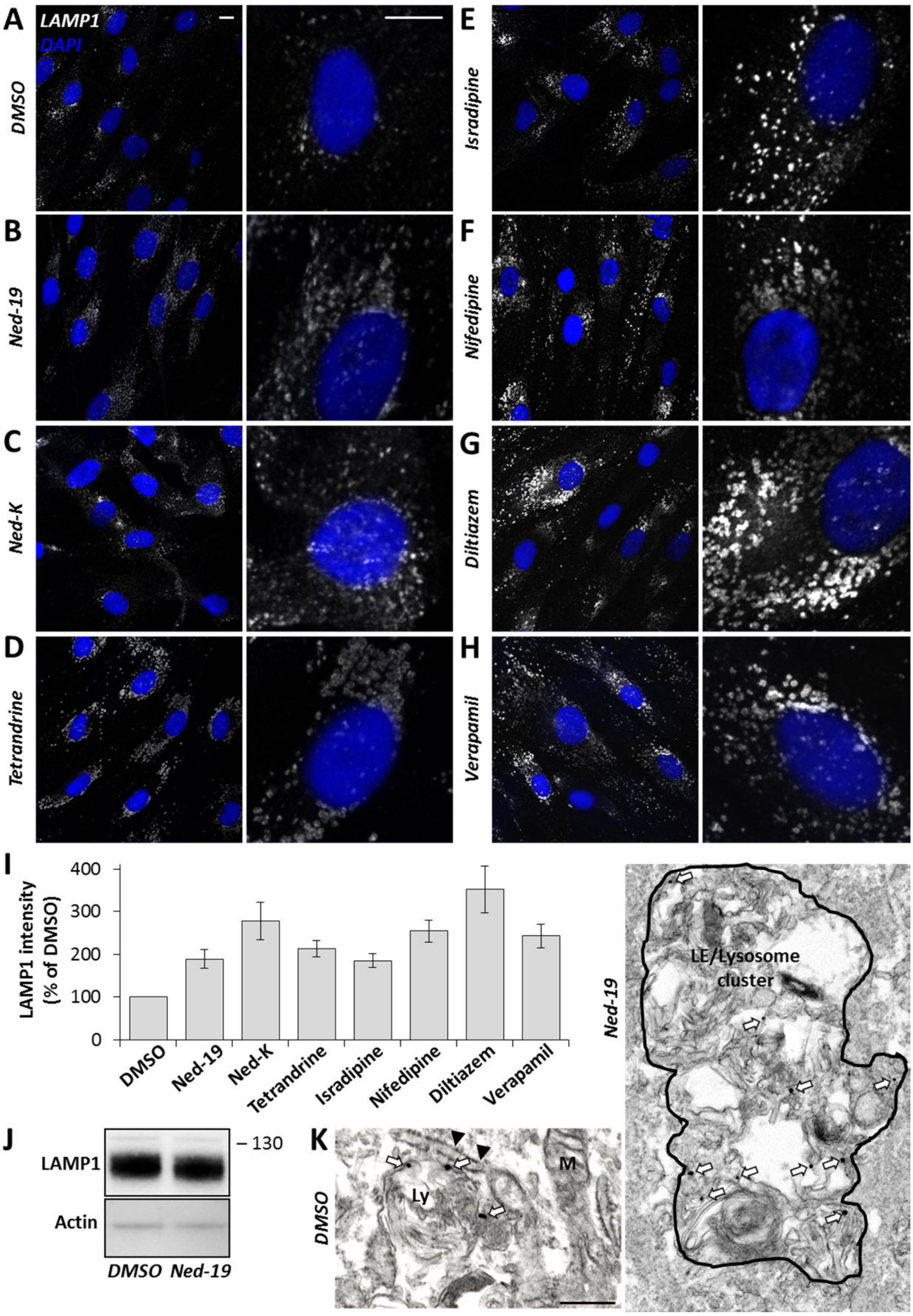

Figure S1. TPCs maintain late endosome/lysosome morphology, Related to Figure 1.

(A-H) Representative confocal images of LAMP1 staining (white) in fibroblasts treated with either DMSO (0.1%; A), the NAADP antagonists Ned-19 (100 µM, B) and Ned-K (100 µM, C), or the Ca^{2+} channel blockers tetrandrine (10 µM, D), isradipine (100 µM, E), nifedipine (100 µM, F), diltiazem (100 µM, G) and verapamil (100 µM, H) for 2h. Nuclei were stained using DAPI (blue). Zoomed images are displayed in the right panels. Scale bars, 10 µm.

(I) Summary data quantifying LAMP1 intensity as a percentage of DMSO control. Data from 39-49 cells from 3 independent platings.

(J) Representative Western blot using antibodies to LAMP1 (top) or actin (bottom) and homogenates from fibroblasts treated with Ned-19 (100 µM) overnight.

(K) Representative electron micrographs of LAMP1 (white arrows) labelling in fibroblasts treated with either DMSO (0.1%) or Ned-19 (100 µM). Typical cluster of LAMP1-positive late endosome/lysosomes is outlined in black. Ly, lysosome; M, mitochondria; black arrowheads, ER. Scale bar, 300nm.

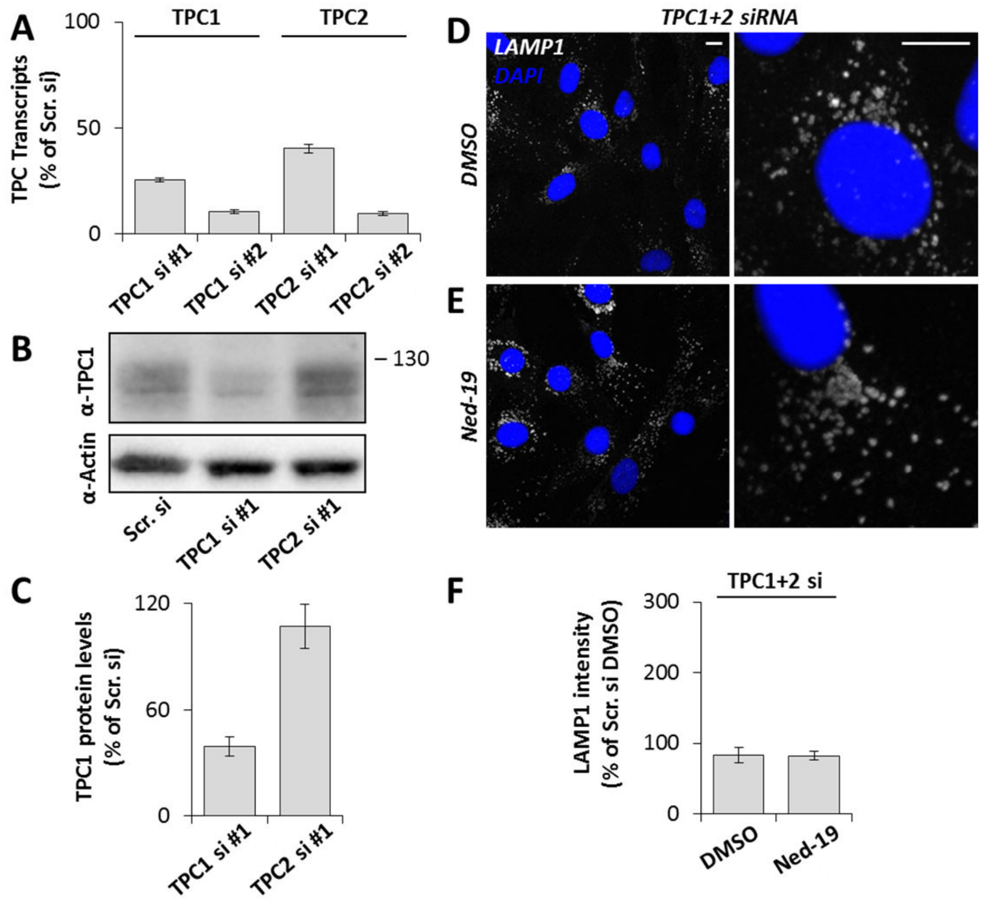

Figure S2. Knockdown of TPCs, Related to Figure 2.

(A) Quantitative PCR analysis of TPC1 (left) and TPC2 (right) levels in fibroblasts transfected with the indicated TPC siRNA for 48h. Data are from 1-4 experiments (analysing 1-2 fibroblast lines) and normalised to TPC levels in cells transfected with scrambled (Scr) siRNA.

(B) Representative Western blot using antibodies to TPC1 (top) or actin (bottom) and homogenates from fibroblasts treated with the indicated siRNA.

(C) Summary data quantifying TPC1 protein levels from 6 independent knockdowns. Data are normalised to TPC1 levels in cells treated with scrambled siRNA.

(D-E) Representative LAMP1 staining (white) in fibroblasts co-transfected with siRNAs targeting TPC1 and TPC2 for 48h and treated for the last 18h with either DMSO (0.1%, D) or Ned-19 (100 µM, E). Nuclei were stained using DAPI (blue). Zoomed images are displayed in the right panels.

(F) Summary data quantifying LAMP1 intensity as a percentage of DMSO. Data are from 39-40 cells from 3 independent knock-downs.

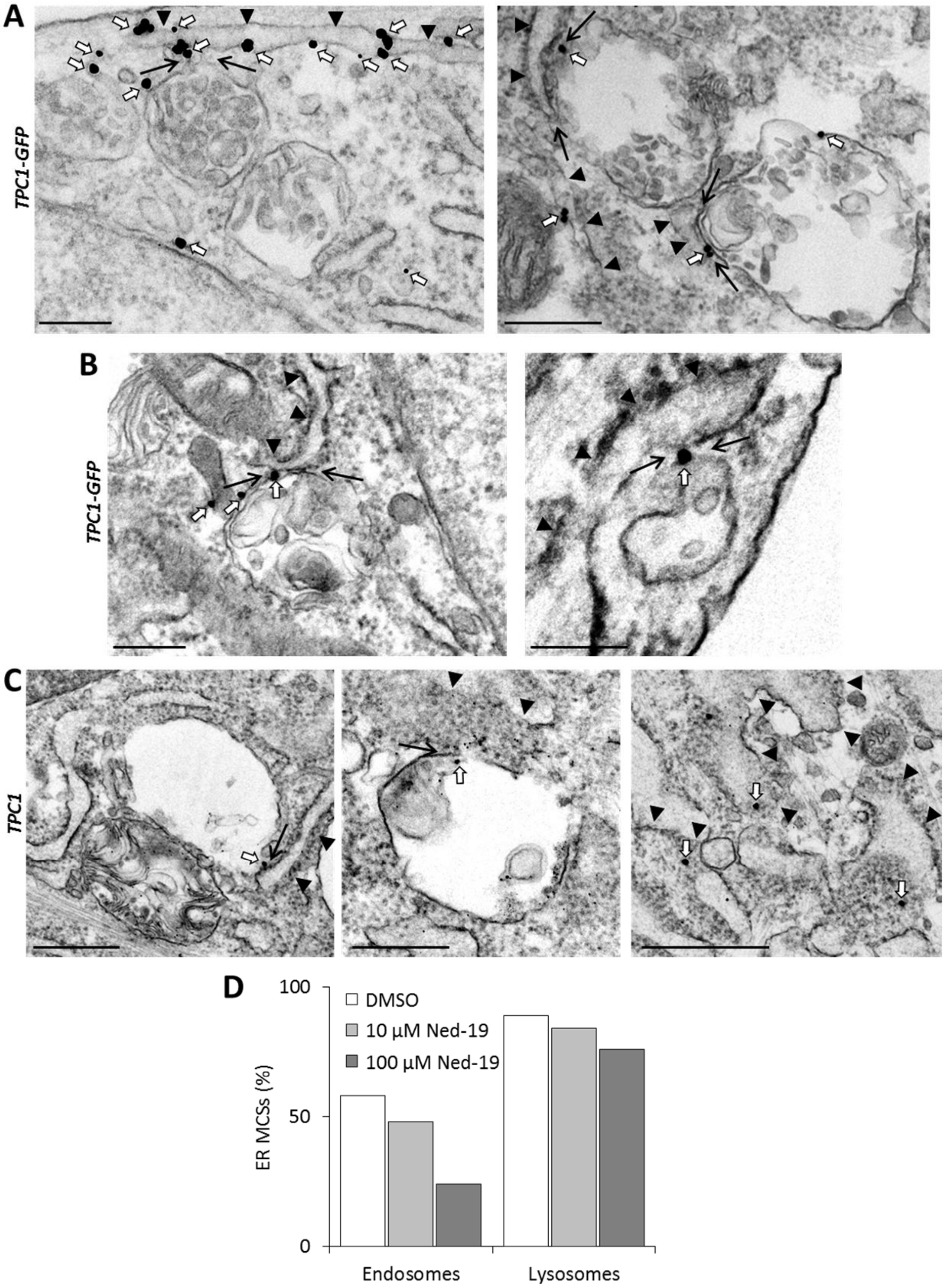

Figure S3. TPC1 localises to ER-endosome contact sites and regulates their formation, Related to Figure 4.

(A-C) Electron micrographs showing distribution of TPC1 in fibroblasts. Cells were transfected with TPC1-GFP (A-B) or untagged TPC1 (C) and stained for GFP/TPC1 using pre-embedding labelling. TPC1 (white arrows) partially localises to the ER (arrowheads) as well as endosomes and to ER-endosome membrane contact sites (black arrows). Scale bar, 200 nm.

(D) Analysis of ER-endosome or lysosome membrane contact sites in Hela cells treated with DMSO (0.1%) or Ned-19 (10 or 100 µM) overnight.

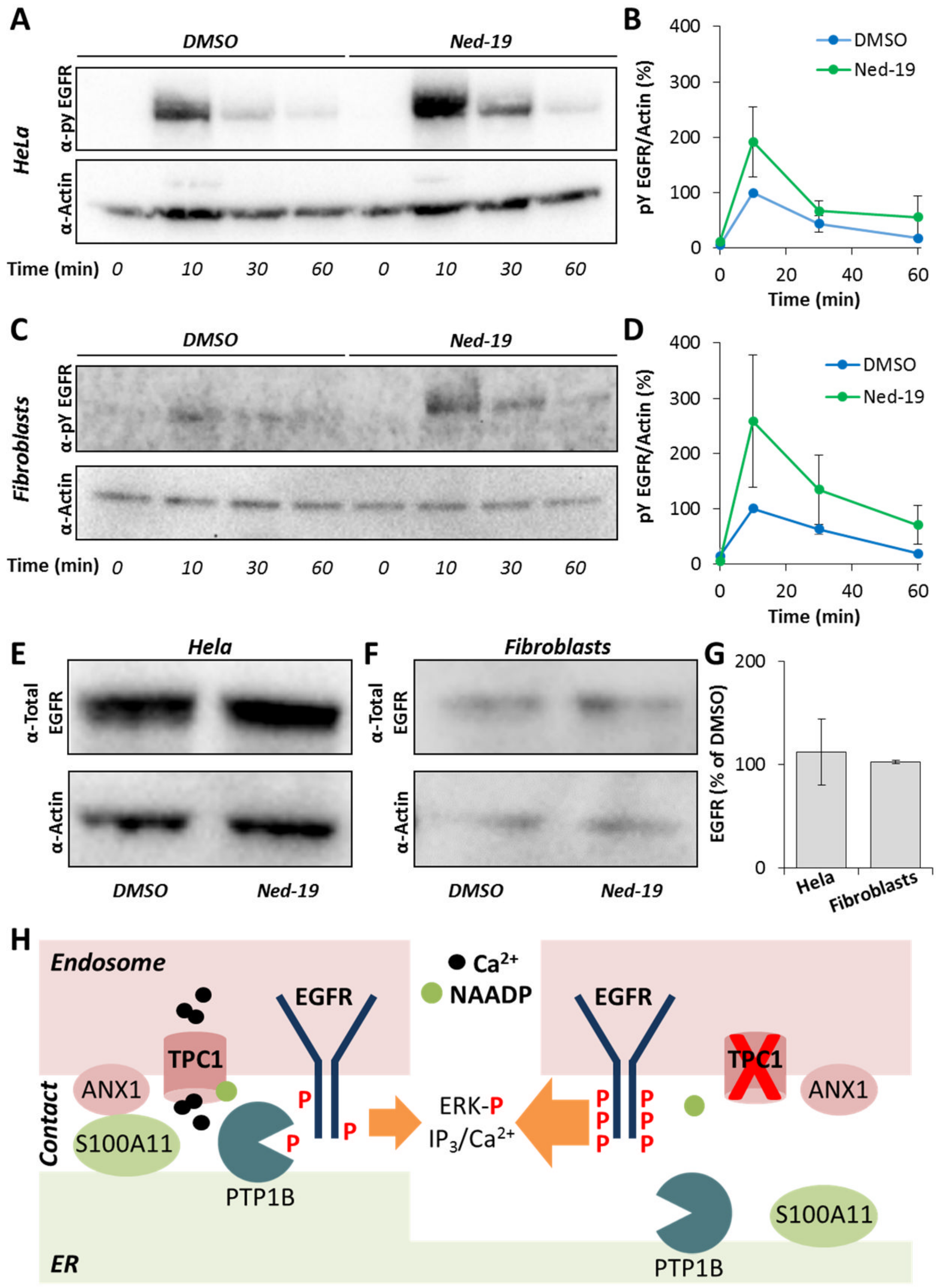

Figure S4. NAADP regulates EGF signalling, Related to Figure 5.

(A-D) Western blot using antibodies to phosphotyrosine (pY) 1068 EGFR (top) or actin (bottom) and homogenates from Hela cells (A) or fibroblasts (C) treated with either DMSO (0.1%) or Ned-19 (100 µM) overnight and serum starved for 1h prior to EGF (100 ng/mL) stimulation at the indicated times. Summary data analysing phosphotyrosine-EGFR receptor levels (normalised to actin) in lysates from Hela cells (B) or fibroblasts (D) quantified as a percentage of DMSO control 10 minutes after EGF stimulation. Data are from 3 independent treatments.

(E-F) Western blot using antibodies to total EGFR (top) or actin (bottom) and homogenates from Hela cells (E) or fibroblast (F) treated with either DMSO (0.1%) or Ned-19 (100 µM) overnight.

(G) Summary data analysing total EGFR receptor levels in lysates quantified as a percentage of DMSO control. Data are from 3-4 independent treatments.

(H) Left, schematic depicting local NAADP-dependent Ca^{2+} flux through TPC1 at membrane contact sites between the endosomes and ER. These sites are populated by ANX1/S100A11 and the ER-localised protein phosphatase PTP1B which dephosphorylates endocytosed EGFRs during stimulation thereby limiting signalling through downstream pathways such as ERK and PLCγ (small arrow). Right, disrupting NAADP/TPC1 signalling (red cross) reduces contact resulting in greater phosphorylation of EGF receptors and enhanced signalling (large arrow).

Treatment	Time	Endo-lysosome size (μm^2)	n
DMSO (0.1 % v/v)	Overnight	0.42 ± 0.005	5256
Ned-19 (100 µM)	Overnight	0.47 ± 0.007	4222
Ned-K (100 µM)	Overnight	0.53 ± 0.009	6734
Tetrandrine (10 µM)	Overnight	0.55 ± 0.08	3892
DMSO (0.1 % v/v)	2h	0.36 ± 0.005	4379
Ned-19 (100 µM)	2h	0.42 ± 0.005	8415
Ned-K (100 µM)	2h	0.45 ± 0.006	6734
Tetrandrine (10 µM)	2h	0.65 ± 0.01	3892
Isradipine (100 µM)	2h	0.45 ± 0.001	7489
Nifedipine (100 µM)	2h	0.47 ± 0.006	6796
Diltiazem (100 µM)	2h	0.51 ± 0.008	6883
Verapamil (100 µM)	2h	0.52 ± 0.007	5537
Control siRNA	72h	0.40 ± 0.007	14600
TPC1 siRNA	72h	0.43 ± 0.005	29199
TPC2 siRNA	72h	0.39 ± 0.004	19663

Table S1. NAADP/TPC signalling maintains late endosome/lysosomal morphology, Related to Figures 1 and 2.

Effect of the indicated drug or siRNA treatment for the given time on late endosome/lysosome size. The area of the indicated number (n) of LAMP1-labelled structures were quantified from confocal micrographs from 3 independent experiments.
