# Supplementary material for: An Endosomal NAADP-Sensitive Two-Pore Ca2+ Channel Regulates ER-Endosome Membrane Contact Sites to Control Growth Factor Signaling
Source: Cell Rep. 2017 Feb 14;18(7):1636–45. doi: 10.1016/j.celrep.2017.01.052 (PMC5318655; doi:10.1016/j.celrep.2017.01.052)
Supplement: Document S2. Article plus Supplemental Information [file mmc2.pdf]

Cell Reports

Report

An Endosomal NAADP-Sensitive Two-Pore Ca^{2+} Channel Regulates ER-Endosome Membrane Contact Sites to Control Growth Factor Signaling

Graphical Abstract

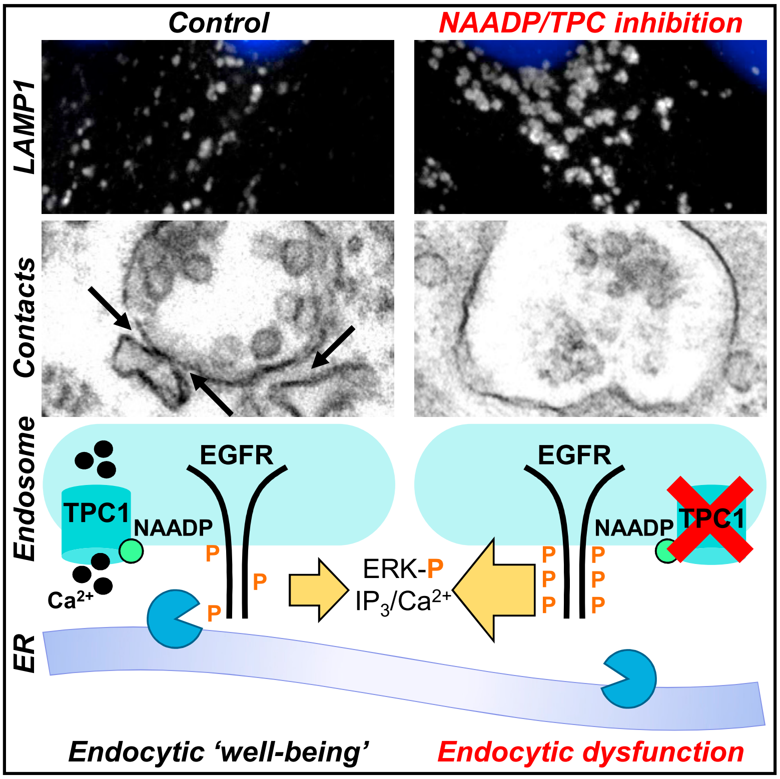

Highlights

- NAADP/TPC1 signaling maintains endo-lysosomal morphology
- TPC1 localizes to contacts between late endosomes and the endoplasmic reticulum
- NAADP/TPC1 signaling regulates contact site formation
- NAADP tempers EGF receptor-mediated signaling

Authors

Bethan S. Kilpatrick, Emily R. Eden, Leanne N. Hockey, Elizabeth Yates, Clare E. Futter, Sandip Patel

Correspondence

c.futter@ucl.ac.uk (C.E.F.),
patel.s@ucl.ac.uk (S.P.)

In Brief

Endosomes form junctions with the ER, but how this contact is regulated remains unclear. Kilpatrick et al. find that Ca^{2+} release by an endosomal ion channel facilitates inter-organellar coupling to temper signals mediated by an internalized growth factor receptor. Endosome-ER contact sites thus emerge as Ca^{2+}-dependent signaling hubs.

Kilpatrick et al., 2017, Cell Reports *18*, 1636–1645
February 14, 2017 © 2017 The Author(s).
http://dx.doi.org/10.1016/j.celrep.2017.01.052

CellPress

OPEN ACCESS
CellPress

Cell Reports
Report

An Endosomal NAADP-Sensitive Two-Pore Ca^{2+} Channel Regulates ER-Endosome Membrane Contact Sites to Control Growth Factor Signaling

Bethan S. Kilpatrick,[1,3] Emily R. Eden,[2,3] Leanne N. Hockey,[1] Elizabeth Yates,[1] Clare E. Futter,[2,*] and Sandip Patel[1,4,*]
[1]Department of Cell and Developmental Biology, University College London, London WC1E 6BT, UK
[2]Department of Cell Biology, Institute of Ophthalmology, University College London, London EC1V 9EL, UK
[3]Co-first author
[4]Lead Contact
*Correspondence: c.futter@ucl.ac.uk (C.E.F.), patel.s@ucl.ac.uk (S.P.)
http://dx.doi.org/10.1016/j.celrep.2017.01.052

SUMMARY

Membrane contact sites are regions of close apposition between organelles that facilitate information transfer. Here, we reveal an essential role for Ca^{2+} derived from the endo-lysosomal system in maintaining contact between endosomes and the endoplasmic reticulum (ER). Antagonizing action of the Ca^{2+}-mobilizing messenger NAADP, inhibiting its target endo-lysosomal ion channel, TPC1, and buffering local Ca^{2+} fluxes all clustered and enlarged late endosomes/lysosomes. We show that TPC1 localizes to ER-endosome contact sites and is required for their formation. Reducing NAADP-dependent contacts delayed EGF receptor de-phosphorylation consistent with close apposition of endocytosed receptors with the ER-localized phosphatase PTP1B. In accord, downstream MAP kinase activation and mobilization of ER Ca^{2+} stores by EGF were exaggerated upon NAADP blockade. Membrane contact sites between endosomes and the ER thus emerge as Ca^{2+}-dependent hubs for signaling.

INTRODUCTION

How organelles communicate is a fundamental question that arises given the compartmentalized nature of eukaryotic cell function. Although vesicular traffic is an established means of information transfer, it is becoming clear that traffic also proceeds by non-vesicular means. In particular, membrane contact sites have emerged as potential platforms for both Ca^{2+} signaling and lipid transfer (Helle et al., 2013; Phillips and Voeltz, 2016; Levine and Patel, 2016; Eden, 2016). Membrane contact sites are regions of close apposition between membranes that are stabilized by tethering complexes. The endoplasmic reticulum (ER) forms multiple classes of contacts with both the plasma membrane and organelles such as endosomes, lysosomes, and mitochondria. Endosome-ER contacts have been implicated in endosome positioning (Rocha et al., 2009; Raiborg et al., 2015a), dephosphorylation of internalized receptors, and components of the endosomal sorting complex required for transport (ESCRT) machinery (Eden et al., 2010, 2016; Stuible et al., 2010), endosome fission (Rowland et al., 2014), actin nucleation and retromer-dependent budding (Dong et al., 2016), and cholesterol transport (Eden et al., 2016). We have identified multiple populations of contact sites that form between the ER and different endocytic organelles (Eden et al., 2016), which include those dependent on VAPs (Dong et al., 2016). Notably, contact sites between the ER and EGF receptor-containing endosomes require annexin-A1 and its Ca^{2+}-dependent binding partner S100A11 (Eden et al., 2016), raising the possibility that Ca^{2+} fluxes may regulate contact.

Ca^{2+} is a widespread signaling ion regulating a range of cellular processes including aspects of vesicle formation, fusion, and traffic (Berridge et al., 2003). Ca^{2+} signals often invade the cell entirety (global) but they can also be spatially restricted (local), as exemplified by signals generated by the Ca^{2+}-mobilizing messenger, nicotinic acid adenine dinucleotide phosphate (NAADP) (Galione, 2015). NAADP is unusual in mediating Ca^{2+} release from the endo-lysosomal system, an acidic Ca^{2+} store filled by Ca^{2+}/H^{+} exchange (Churchill et al., 2002; Patel and Muallem, 2011; Melchionda et al., 2016). It does so by activating two-pore channels (TPCs) (Calcraft et al., 2009; Brailoiu et al., 2009; Patel, 2015). Local NAADP-mediated Ca^{2+} release events from acidic organelles are amplified by Ca^{2+} channels on canonical Ca^{2+} stores of the ER to generate global signals (Galione, 2015). This occurs during signaling by external cues such as hormones and neurotransmitters (Yamasaki et al., 2005; Pandey et al., 2009). However, it is also evident that local TPC-mediated Ca^{2+} release events function in a constitutive manner. For instance, NAADP/TPC signaling regulates several membrane trafficking events, including retrograde traffic from endosomes to the Golgi (Ruas et al., 2010, 2014) and the trafficking of cholesterol, receptors, and viruses (Grimm et al., 2014; Ruas et al., 2014; Sakurai et al., 2015). This pathway also regulates endo-lysosomal morphology (Lin-Moshier et al., 2014; Hockey et al., 2015; Patel, 2015), likely through Ca^{2+}-dependent vesicular fusion/fission events (Pryor et al., 2000; Luzio et al., 2007; Marchant and Patel, 2015). However, what role TPCs play in non-vesicular trafficking is unexplored (Burgoyne et al., 2015).

 © 2017 The Author(s).
This is an open access article under the CC BY license (http://creativecommons.org/licenses/by/4.0/).

Here, we reveal an essential requirement for NAADP and TPC1 in regulating membrane contact site formation between endosomes and the ER to control growth factor signaling.

RESULTS

NAADP and TPC1 Maintain Late Endosome and Lysosome Morphology

We examined the effect of inhibiting NAADP action on late endosome and lysosome morphology in primary human fibroblasts using four approaches.

First, we tested NAADP antagonists. Figures 1A and 1B show the effect of an overnight treatment with Ned-19 (Naylor et al., 2009) on late endosome and lysosome morphology as assessed by immuno-fluorescence staining and confocal microscopy of the late endosome and lysosome marker LAMP1. Labeled structures were clustered in the perinuclear region and often appeared enlarged (Figure 1B; changes in staining intensity quantified in Figure 1H). Similar results were obtained with the recently described Ned-19 analog, Ned-K (Hockey et al., 2015; Davidson et al., 2015) (Figure 1C) and upon shorter (2-hr) treatment with the antagonists (Figures S1A–S1C and S1I). Analysis of multiple individual labeled structures revealed an increase in the mean area (Table S1). LAMP1 protein levels were similar upon Ned-19 treatment (Figure S1J). We further examined the ultrastructure of the endo-lysosomal system by electron microscopy (EM). Consistent with results using light microscopy, late endosomes and electron-dense lysosomes were often clustered and more vacuolar in Ned-19-treated cells compared with controls (Figure 1D; quantified in Figure 1I). Immuno-EM confirmed that LAMP1 localizes to late endosome and lysosome clusters in Ned-19-treated cells (Figure S1K).

Second, we pharmacologically targeted the TPC pore. Recent studies have shown that TPCs are inhibited by the plant alkaloid tetrandrine (Sakurai et al., 2015). Like NAADP antagonists, tetrandrine clustered late endosome and lysosomes and induced particularly pronounced vesicle enlargement (Figures 1E, 1J, S1D, and S1I; Table S1). TPCs have also emerged as targets for L-type Ca^{2+} channel blockers (Rahman et al., 2014). Therefore, we examined the effect of isradipine and nifedipine (both dihydropyridines), diltiazem (a benzothiazapine), and verapamil (a phenylalkylamine). As shown in Figures S1E–S1I and Table S1, all three structurally distinct classes of inhibitors induced aggregation/vesicle enlargement following an acute treatment.

Third, we buffered Ca^{2+} levels. Treatment of cells with the cell-permeable form of the Ca^{2+} chelator, 1,2-bis(*o*-aminophenoxy) ethane-*N*,*N*,*N*′,*N*′-tetraacetic acid (BAPTA), also altered the appearance of late endosome and lysosomes (Figure 1F). In contrast, treatment with the slower Ca^{2+} chelator EGTA did not (Figure 1G). This differential sensitivity to chelators (Stern, 1992) summarized in Figure 1J suggests that morphology of the endo-lysosomal system is regulated by local Ca^{2+} fluxes.

Fourth, to directly probe the role of TPCs in endo-lysosomal morphology, we examined the effect of TPC knockdown. Treatment of fibroblasts with small interfering RNAs (siRNAs) targeting two independent sequences in both TPC1 or TPC2 reduced transcript levels >50% (Figure S2A). Knockdown of TPC1 protein was confirmed by western blotting (Figures S2B and S2C). As shown in Figures 2A–2C, confocal microscopy of TPC1-silenced cells revealed marked changes in LAMP1 staining (quantified in Figure 2F and Table S1), similar to chemical blockade of NAADP signaling (Figure 1). TPC2 silencing, however, had little effect (Figures 2D and 2E). In accord, late endosome and lysosomes appeared more clustered and less distinct upon TPC1, but not TPC2, silencing at the ultrastructural level (Figures 2G–2K, quantified in Figure 2L). To assess specificity of our molecular manipulations, we performed rescue experiments with a siRNA-resistant TPC1 construct. Expression of this construct in TPC1-depleted cells partially reversed clustering of late endosome and lysosomes as assessed by both LAMP1 immunocytochemistry (Figures 3A and 3B) and correlative light and electron microscopy (CLEM) (Figures 3C and 3D), thereby attesting to specificity. Late endosome and lysosomal morphology was also unchanged by Ned-19 in cells where both TPC1 and TPC2 had been silenced (Figures S2D–S2F), further attesting to specificity. Late endosome and lysosomal form is thus specifically determined by TPCs in an isoform-selective manner.

Taken together, we identify an unexpected role for NAADP, a target channel, and associated local Ca^{2+} fluxes in maintaining late endosome and lysosomal morphology.

TPC1 Localizes to Endosome-ER Contact Sites and Regulates Their Formation

The distribution of TPC1 within the endocytic pathway is unclear and likely more diffuse than that of TPC2, which is expressed predominantly on lysosomes (Brailoiu et al., 2009). Immuno-EM revealed localization of GFP-tagged or untagged TPC1 to the limiting membrane of multi-vesicular endosomes, rather than electron-dense lysosomes, with some additional ER localization that might be related to ectopic expression (Figures 4A and S3A–S3C). Parallel studies with TPC2, however, were ambiguous because expression of TPC2 resulted in a proliferation of endocytic organelles with disorganized membranous content, often aggregated in clusters (Figure 4A). TPC2-mediated disruption of late endosome-lysosomal morphology is consistent with our previous analysis (Lin-Moshier et al., 2014). Intriguingly, we found that TPC1 was often found at membrane contact sites between endosomes and the ER (Figures 4B and S3A–S3C). Quantitative analysis of TPC1-positive endosomes showed that there was a ~5-fold increase in the number of gold particles/unit endosomal membrane in contact with the ER compared to regions not associated with the ER (Figure 4B). TPC1 thus emerges as a component of ER-endosome contacts.

The presence of TPC1 at ER-endosome contact sites raised the possibility that local Ca^{2+} signals deriving from the endosome may regulate contact with the ER. We therefore examined the effect of NAADP blockade on ER-endosome contact site formation (Figure 4C). As shown in Figure 4D, Ned-19 reduced the percentage of endosomes with an ER contact. Similar results were obtained in HeLa cells where Ned-19 reduced ER-endosome contact site formation in a concentration-dependent manner (Figure S3D). The effects of Ned-19 were recapitulated by knocking down TPC1, whereas depletion of TPC2 had little effect on ER-endosome contacts in fibroblasts

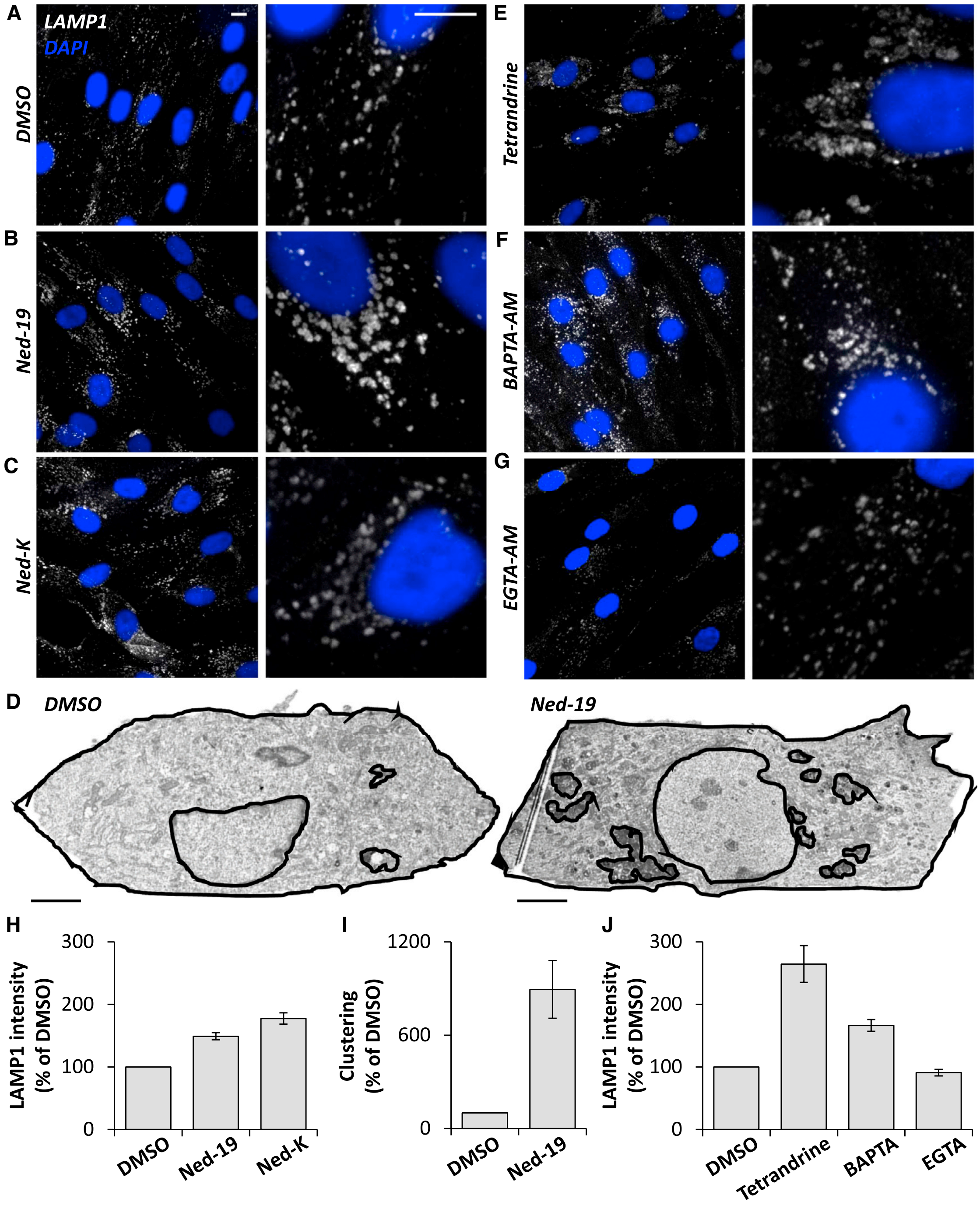

Figure 1. NAADP Signaling Maintains Late Endosome and Lysosomal Morphology

(A–C) Representative confocal images of LAMP1 staining (white) in fibroblasts treated overnight with either DMSO (0.1%) (A) or the NAADP antagonists, Ned-19 (100 μM) (B) and Ned-K (100 μM) (C). Nuclei were stained using DAPI (blue). Zoomed images are displayed in the right panels. Scale bars, 10 μm.

(D) Representative electron micrographs of the endo-lysosomal system in fibroblasts treated overnight with either DMSO (0.1%) (left) or Ned-19 (100 μM) (right). Regions of interest corresponding to endo-lysosome clusters, the nucleus, and the cell entirety are highlighted. Scale bars, 5 μm.

(E–G) LAMP1 staining in fibroblasts treated overnight with the TPC blocker, tetrandrine (Tet, 10 μM) (E) or for 2 hr with acetoxymethyl (AM) esters of the Ca^{2+} chelators BAPTA (50 μM) (F) or EGTA (50 μM) (G).

(H–J) Summary data quantifying LAMP1 intensity in cells treated with NAADP antagonists (H) (95–299 cells from 3–11 independent treatments), clustering of endo-lysosomes in cells treated with Ned-19 expressed as a percentage of the area occupied relative to non-nuclear cytoplasm (I) (ten cells under each

(legend continued on next page)

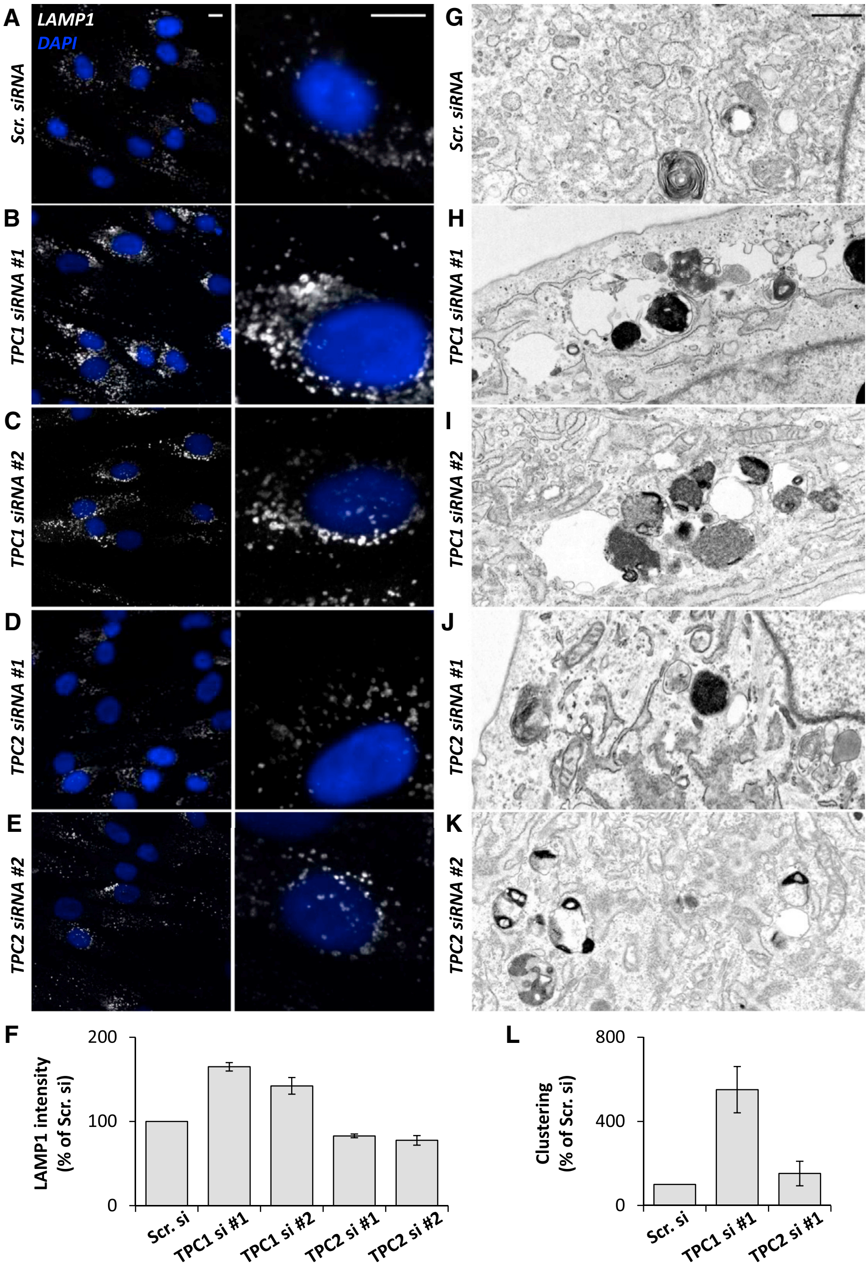

Figure 2. TPC1 Maintains Late Endosome and Lysosomal Morphology

(A–E) Representative LAMP1 staining (white) in fibroblasts transfected with scrambled (Scr) siRNA (A) or with two independent siRNAs targeting TPC1 (B and C) or TPC2 (D and E) for 48 hr. Nuclei were stained using DAPI (blue). Zoomed images are displayed in the right panels. Scale bars, 10 μm.

(F) Summary data quantifying LAMP1 intensity as a percentage of scrambled control (± SEM). Data are from 57–496 cells from 3–11 independent knockdowns.

(G–K) Representative electron micrographs of the endo-lysosomal system in fibroblasts transfected with Scr siRNA (G) or with siRNAs targeting TPC1 (H–I) or TPC2 (J–K). Scale bar, 500 nm.

(L) Summary data quantifying clustering of endo-lysosomes expressed as a percentage of the area occupied relative to non-nuclear cytoplasm. Data are from ten cells under each condition and normalized relative to Scr siRNA (± SEM).

See also Figure S2 and Table S1.

(Figure 4D). Chemical and molecular inhibition of NAADP signaling on contact site formation thus mirrors the effect on gross late endosome and lysosomal morphology (Figures 1 and 2).

The ER forms contacts with lysosomes (Kilpatrick et al., 2013) that are biochemically distinct from those with endosomes (Eden et al., 2016). To assess specificity of our manipulations, we examined the effect of NAADP blockade on ER-lysosome contact site formation (Figure 4C). Ned-19 had little effect on the percentage of lysosomes with an ER contact in both fibroblasts (Figure 4E) and HeLa cells (Figure S3D). Silencing of TPC1 was also largely without effect, whereas silencing of TPC2 reduced ER-lysosome contact sites (Figure 4E).

In summary, these data uncover a highly isoform- and compartment-specific role for NAADP signaling in the formation of membrane contacts between endosomes and the ER.

NAADP Regulates EGF Signaling

We have shown previously that ER-endosome contacts enable the interaction between endocytosed EGF receptor (EGFR) tyrosine kinase and the protein tyrosine phosphatase, PTP1B, on the ER (Eden et al., 2010). This contact allows receptor de-phosphorylation—a determinant of signaling by EGF. We reasoned that disrupting NAADP signaling would enhance EGF signaling due to compromised contact at the ER-endosome interface. To test this, we examined the effect of Ned-19 on the phosphorylation state of EGFR. Acute stimulation with EGF induced a transient rise in EGFR tyrosine phosphorylation that was significantly enhanced and prolonged by Ned-19 treatment (Figure 5A, quantified in Figures 5B, S4A, and S4B). For these experiments, we used HeLa cells due to their high EGFR levels, but similar results were obtained in fibroblasts (Figures S4B–S4D).

condition), and LAMP1 intensity in cells treated with tetrandrine/Ca^{2+} chelators (J) (73–116 cells from three to five independent treatments). Data are presented as a percentage of DMSO controls (±SEM).
See also Figure S1 and Table S1.

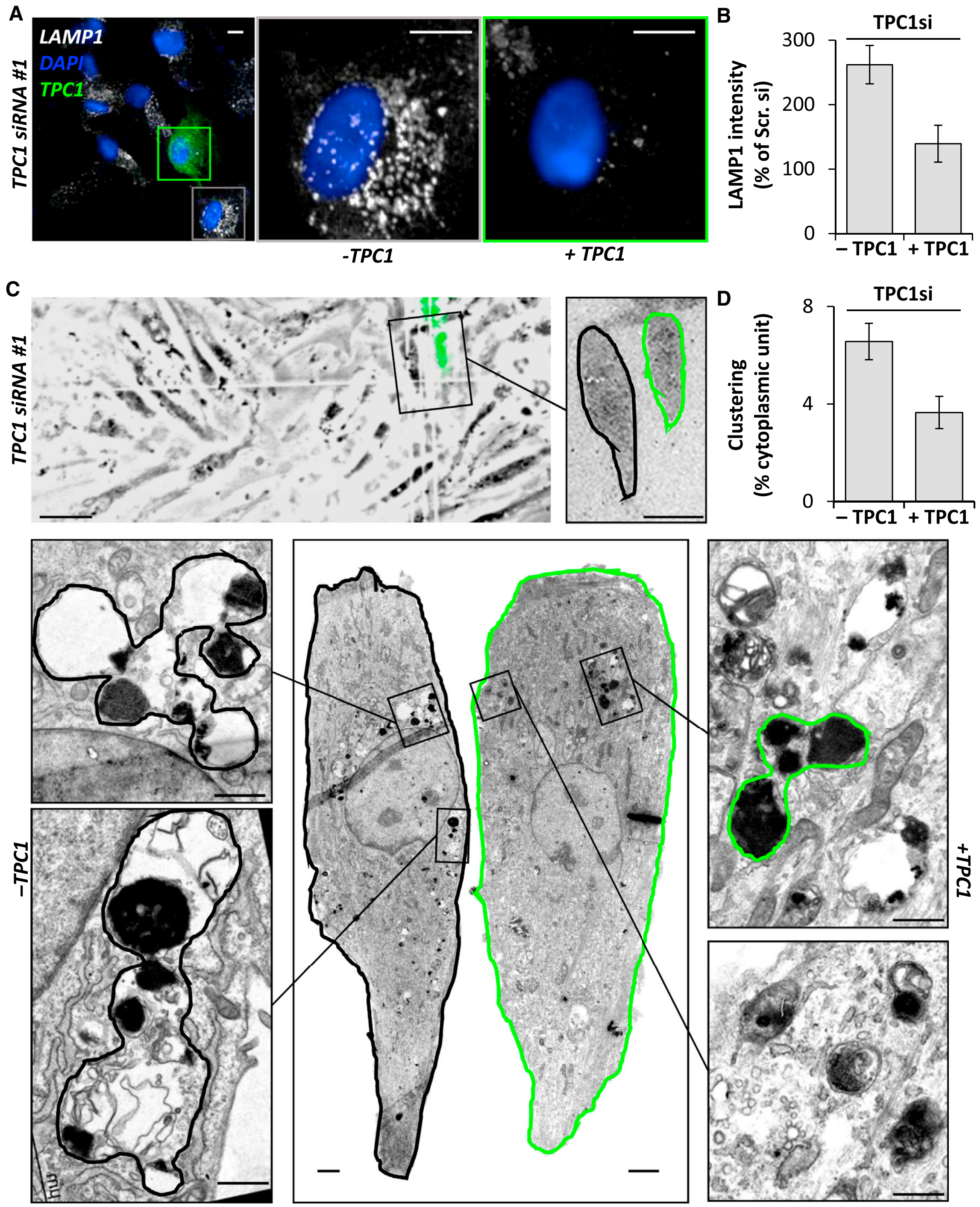

Figure 3. TPC1-Mediated Late Endosome and Lysosome Morphology Defects Are Rescuable

(A) Representative LAMP1 staining (white) in fibroblasts transfected with TPC1 siRNA#1 and GFP-tagged siRNA-resistant TPC1 (green). Nuclei were stained using DAPI (blue). Zoomed images of cells that were negative (gray box) and positive (green box) for GFP are displayed in the right panels. Scale bars, 10 μm.

(B) Summary data quantifying LAMP1 intensity as a percentage of scrambled control (± SEM). Data are from 18–30 cells from two independent knockdowns.

(C) Representative CLEM of cultures similar to (A) with light microscopy images shown in the top panel (scale bar, 50 μm) from which a pair of cells (black blox; scale bar, 20 μm) were identified for EM. Bottom panels show cropped EM images of the cells in their entirety (center; scale bars, 2 μm) and zoomed images of the indicated region from the cell that was GFP-negative (left) and GFP-positive (right; scale bars, 1 μm).

(D) Summary data quantifying clustering of late endosomes/lysosomes expressed as a percentage of the area occupied relative to non-nuclear cytoplasm (± SEM). Data are from four cells under each condition.

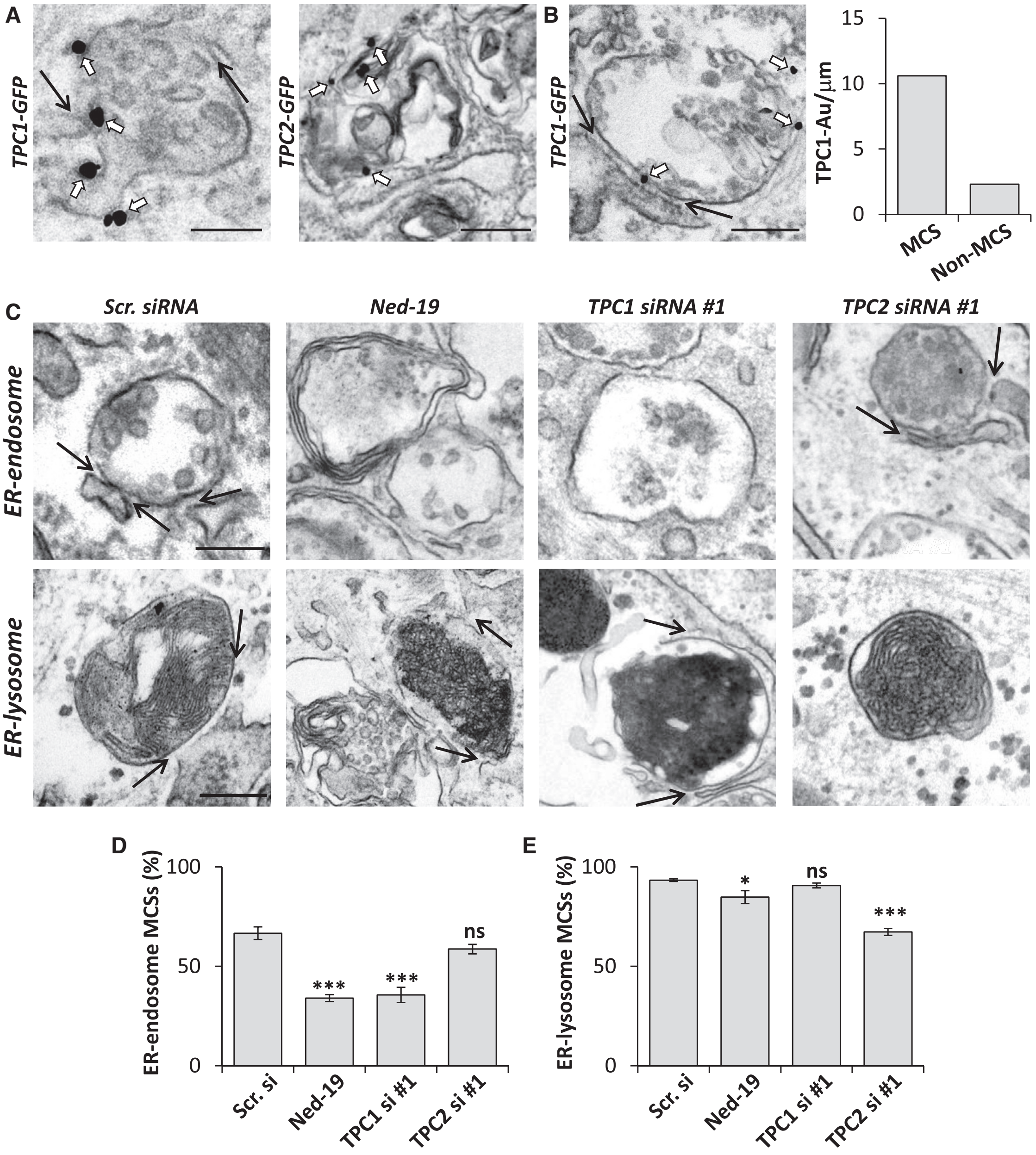

Figure 4. TPC1 Localizes to ER-Endosome Contact Sites and Regulates Their Formation

(A and B) Electron micrographs showing distribution of TPC1 and TPC2 in fibroblasts (A) and localization of TPC1 at contact sites (B). Summary data quantifying the presence of TPC1 at contact versus non-contact sites are shown to the right. Cells were transfected with GFP-tagged TPC constructs and stained for GFP (white arrows) using pre-embedding labeling. Scale bar, 200 nm. Black arrows, ER contact sites.

(C–E) Electron micrographs showing ER membrane contact sites between late endosomes and lysosomes in fibroblasts treated with a scrambled siRNA, 100 μM Ned-19, or with siRNA targeting TPC1 or TPC2 (C). The percentage of endosomes (D) or lysosomes (E) with an ER contact site were quantified. Data are from three independent treatments (± SEM). Scale bar, 200 nm. Black arrows, ER contact sites.

See also Figure S3.

To examine downstream functional consequences of disrupting contacts, we took two approaches.

First, we measured ERK activity because activated EGFRs are classically coupled to the MAP kinase pathway. Consistent with the prolonged EGFR activation observed in cells treated with Ned-19, tyrosine phosphorylation of ERK1/2 was significantly increased and extended upon NAADP inhibition (Figures 5C and 5D).

Second, we measured cytosolic Ca^{2+} levels. Activated EGFRs also couple to phospholipase C-gamma (PLCγ), which

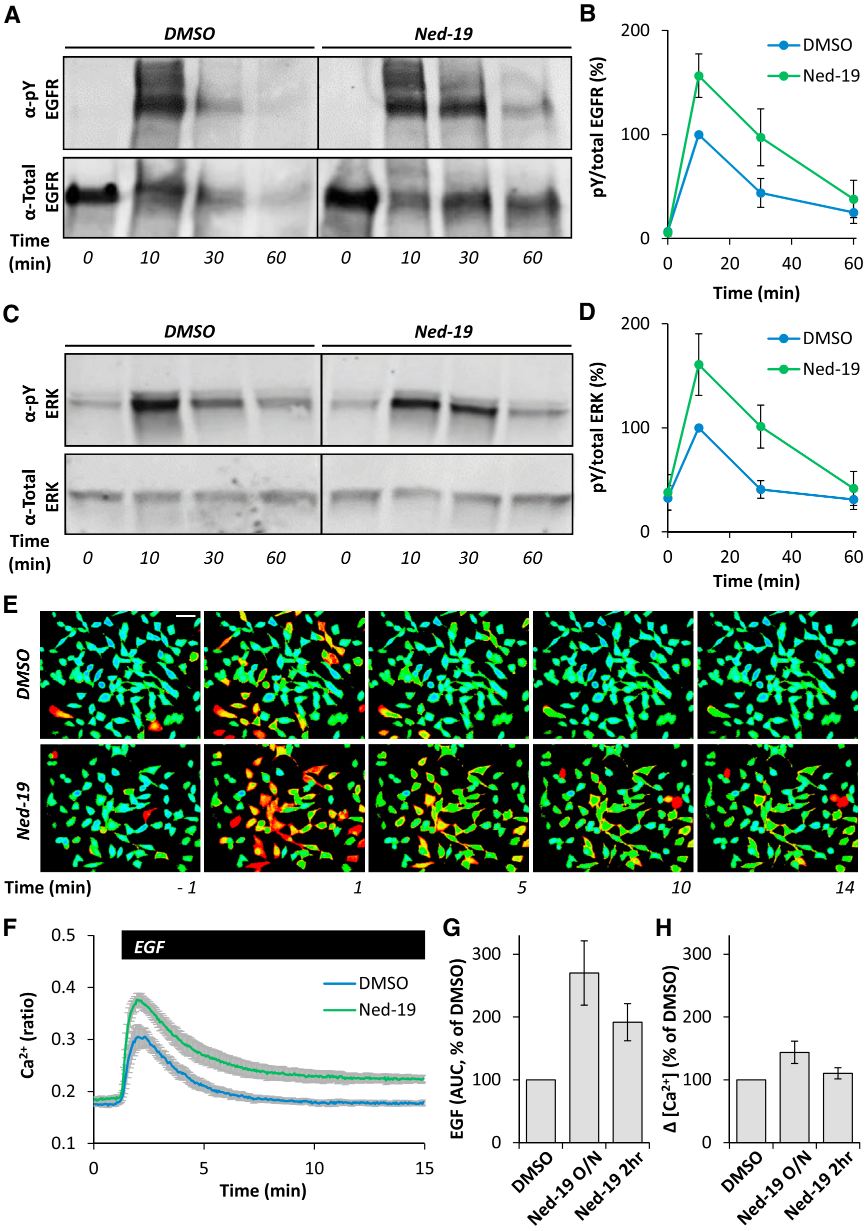

Figure 5. NAADP Regulates EGF Signaling

(A) Representative western blot using antibodies to phosphotyrosine (pY) 1068 EGFR (top) or total EGFR (bottom) and homogenates from HeLa cells. Cells were treated with either DMSO (0.1%) or Ned-19 (100 μM) overnight and serum starved for 1 hr prior to EGF (100 ng/mL) stimulation for the indicated times.

(B) Summary data analyzing pY-EGFR levels (normalized to total EGFR) in lysates quantified as a percentage of DMSO control 10 min after EGF stimulation. Data are from three independent treatments (± SEM).

(C and D) Similar to (A) and (B) except western blots were performed with antibodies to pY 204 ERK1/2 or total ERK1/2 (C). Data are from three independent treatments (± SEM; D).

(E) Pseudo-colored images of the fluorescence ratio of HeLa cells loaded with the Ca^{2+} indicator, Fura-2, and stimulated with EGF (100 ng/mL) for the times indicated. Cells were treated with DMSO (0.1%) or Ned-19 (100 μM) overnight. Scale bar, 50 μm.

(F–H) Summary data quantifying the time course (F), area under the curve (AUC) (G, normalized to DMSO) and maximal change ($\Delta[Ca^{2+}]$) (H, normalized to DMSO) of cytosolic Ca^{2+} levels after stimulation with EGF for the indicated time. Data are from two to four independent treatments (± SEM) analyzing 309–828 cells.

See also Figure S4.

generates inositol 1,4,5-trisphosphate, resulting in Ca^{2+} release from ER Ca^{2+} stores. EGF evoked readily measurable Ca^{2+} signals that were exaggerated and more sustained in cells treated with Ned-19 for 2 hr or overnight (Figures 5E–5H). Total levels of the EGFR were unchanged (Figures S4E–S4G), indicating that enhanced signaling effects are not attributable to increased expression of EGFR. These data are again consistent with prolonged activation of EGFR due to perturbed contact at NAADP-sensitive endosome-ER contact sites.

Taken together, these data reveal a role for NAADP in regulating both EGFR activity and downstream signaling by MAP kinase and phospholipase C.

DISCUSSION

Membrane contact sites between endosomes and the ER are gaining much attention as novel coordinators of cell function (Raiborg et al., 2015b). Contact with the ER is regulated by cholesterol (Rocha et al., 2009) and increases as endocytic vesicles mature (Friedman et al., 2013). Previous studies have shown marked upregulation of contacts in response to expression of STARD3/STARD3NL (Alpy et al., 2013), ORP1L (Rocha et al., 2009), and protrudin (Raiborg et al., 2015a), but whether these proteins are necessary for contact site formation is less clear. Here, we use EM, which, relative to light microscopy, is better suited for resolving inter-organelle junctions to provide direct evidence that TPC1 is a contact site component (Figure 4). Importantly, inhibiting TPC1 activity using chemical and molecular means significantly decreased ER contact site formation with late endosomes (Figure 4). This was associated with disruptions in late endosome and lysosome morphology (Figures 1 and 2), although whether TPC1 and LAMP1 co-localize to junction-forming organelles remains to be established. Consistent with a role for ER-endosome contact sites in endosomal positioning (Rocha et al., 2009; Jongsma et al., 2016), we observed a more perinuclear population of LAMP1-positive late endosome

and lysosomes when contact was reduced by inhibition of NAADP or TPC1 (Figures 1 and 2). We thus provide key evidence implicating endogenous NAADP signaling in regulating ER-endosome contact and the subcellular distribution of endocytic organelles.

Localized Ca^{2+} release from endocytic organelles is a driver of endosome-lysosome fusion (Pryor et al., 2000). In accord, TPCs associate with the fusion apparatus (Lin-Moshier et al., 2014; Grimm et al., 2014) and have been implicated in a number of classical organellar vesicular trafficking events (Marchant and Patel, 2015). Our identification of ER-endosome contact sites dependent on annexin A1 (Eden et al., 2016) and NAADP (this study) establishes a paradigm whereby localized Ca^{2+} release from endocytic organelles might regulate non-vesicular traffic. Of relevance are recent studies showing that Ca^{2+} regulates the formation of contacts between the ER and the plasma membrane (Giordano et al., 2013). Notably, the measured affinity for Ca^{2+} is in the low micromolar range, suggesting that large global signals, such as those evoked during Ca^{2+} influx, regulate these junctions (Idevall-Hagren et al., 2015). However, because of the restricted volume at contacts, it is possible that even modest, constitutive fluxes in unstimulated cells, as alluded to here, could achieve the necessary Ca^{2+} concentrations to modulate Ca^{2+}-dependent contacts. The effects of interfering with NAADP/TPC signaling on contact sites correlated well with effects on gross late endosome and lysosomal morphology. However, possible endosome-lysosome fusion defects might also contribute to morphological changes.

Activated EGF receptors undergo internalization onto endosomes where they continue to signal in their phosphorylated form until they are dephosphorylated by PTP1B on the ER. Disrupting ER-endosome contacts by inhibiting NAADP prolongs EGFR phosphorylation (Figure 5). These data support our findings that EGFR dephosphorylation occurs at endosome-ER contact sites populated by PTP1B and the Ca^{2+}-binding protein annexin A1 (Eden et al., 2010, 2016). Importantly, we also report that disruption of NAADP-dependent contacts substantially enhances downstream signaling by EGF through both ERK and PLCγ (Figure 5). Whether EGF receptors are coupled to NAADP production similar to vascular endothelial growth factor (VEGF) receptors (Favia et al., 2014) is not known at present. Rather our data identify NAADP as a negative regulator of EGF action through local signaling at the endosome-ER interface (Figure S4H). Many external stimuli elevate NAADP levels (Galione, 2015), raising the possibility that second messenger and mitogenic signaling crosstalk may occur through modulation of contact site strength.

In summary, our findings provide new information on the molecular makeup, regulation, and function of ER-endosome contact sites.

EXPERIMENTAL PROCEDURES

Cell Treatments

Primary cultured human fibroblasts and HeLa cells were maintained in DMEM supplemented with 10% (v/v) fetal bovine serum, 100 units/mL penicillin, and 100 μg/mL streptomycin (all from Invitrogen) at 37°C in a humidified atmosphere with 5% CO_2. Cells were passaged by scraping (fibroblasts) or with trypsin (HeLa cells) and plated onto coverslips (for immunocytochemistry, Ca^{2+} imaging, and electron microscopy) or directly onto tissue culture plates/flasks (for western blotting) before experimentation.

For chemical treatments, drugs were dissolved in DMSO or H_2O, diluted into culture medium, and then sterile filtered. The NAADP antagonists, *trans*-Ned-19 and Ned-K were synthesized as described by Naylor et al. (2009) and Davidson et al. (2015), respectively. Both were kind gifts from Raj Gossain, A. Ganesan, and Sean M. Davidson. BAPTA-AM was from Biovision. EGTA-AM was from AnaSpec. Tetrandrine was from Santa Cruz. Isradipine, nifedipine, verapamil, and diltiazem were from Sigma.

Knockdown was performed by transfecting cells with control siRNA duplex (Allstars Negative Control siRNA; QIAGEN) and duplexes targeting human TPC1 (#1, AGCUGUAUUUCAUCAUGAAtt; #2, GGCUACUAUUAUCUCAAUAtt) and TPC2 (#1, GGUGGGACCUCUGCAUUGAtt; #2, CGGUAUUACUCGAACGUAAUtt) (#1, QIAGEN; #2, Ambion) using Lipofectamine RNAiMAX as described previously (Hockey et al., 2015). For overexpression, cells were transfected with untagged TPC1 or C-terminally GFP-tagged human TPC1 and TPC2 as described by Brailoiu et al. (2009), or a rescue construct in which the target sequence of siRNA#1 in TPC1-GFP was mutated to TGAACTCTACTTTATA using the Q5 site-directed mutagenesis kit (NEB) with forward (actttataATGAACCTGCTTCTGGCT) and reverse (agagttcaATGGAGAGGTACACGATG) primers.

Stimulation with EGF (100 ng/mL; Sigma) was performed following serum starvation for 1 hr in serum-free DMEM.

Immunocytochemistry

LAMP1 labeling and confocal microscopy were performed as described by Hockey et al. (2015). Briefly, all images were acquired under identical confocal settings and mean fluorescence intensity per cell quantified to allow comparison between controls and the various treatments. Automated size analysis of labeled structures was performed using the "Analyze Particle" function in Fiji from binary images created by local thresholding using the Bernsen algorithm (5-pixel radius with the default contrast threshold) and Watershed segmentation.

Electron Microscopy

EM was performed essentially as described (Eden et al., 2016). Briefly, serum-starved cells were stimulated with EGF and BSA-gold. After fixation in paraformaldehyde (PFA)/glutaraldehyde, cells were post-fixed in osmium tetroxide/potassium ferricyanide and embedded. Clustering was quantified by calculating the area (using Fiji) of three or more late endosomes/lysosomes in close apposition relative to the cytoplasmic area (excluding the nucleus). For pre-embedding labeling, cells were fixed in PFA, permeabilized with digitonin, and incubated with primary and nanogold-secondary antibodies prior to fixation for EM. ER-endosome contact sites in random sections were defined as regions where apposing membranes were <30 nm apart, with no minimum length. For correlative light and electron microscopy (CLEM), cells were plated on gridded dishes, fixed in 4% PFA, and imaged by light microscopy (Nikon Ti-E), prior to preparation for conventional EM as above.

Ca^{2+} Imaging

Cytosolic Ca^{2+} concentration was measured essentially as described (Kilpatrick et al., 2016). In brief, cells were loaded with the fluorescent Ca^{2+} indicator Fura-2 in HEPES-buffered saline. Dual excitation time-lapse fluorescence imaging was performed using a CCD camera. Data are presented as fluorescence ratios upon excitation at 340 and 380 nm.

Other Methods

Quantitative PCR and western blotting were performed exactly as described by Hockey et al. (2015). The primary antibodies used for western blotting were anti-LAMP1 (mouse; Santa Cruz; 1/500; 1 hr room temperature [RT]), anti-TPC1 (rabbit; Abcam; 1/200; 1 hr RT), anti-actin (goat; Invitrogen; 1/500; 1 hr RT), anti-phosphotyrosine 1068 EGFR (rabbit; Cell Signaling; 1/2,000; 1 hr RT), anti-EGFR (sheep; Fitzgerald; 1/2000; 1 hr RT), anti-phosphotyrosine

204 ERK1/2 (mouse; Santa Cruz; 1/1,000), and anti-ERK1/2 (rabbit; Cell Signaling; 1/1,000).

SUPPLEMENTAL INFORMATION

Supplemental Information includes four figures and one table and can be found with this article online at http://dx.doi.org/10.1016/j.celrep.2017.01.052.

AUTHOR CONTRIBUTIONS

B.S.K. performed the cell culture, knockdowns, and Ca^{2+} imaging. B.S.K. and E.R.E. performed the western blotting. E.R.E. performed the electron microscopy. L.N.H. and B.S.K. performed the drug treatments, immunocytochemistry, and confocal microscopy. B.S.K. and E.Y. performed the qPCR and LAMP1 area analysis. C.E.F. and S.P. conceived and jointly supervised the study. S.P. and B.S.K. wrote the paper with input from all authors.

ACKNOWLEDGMENTS

This work was supported by grants from the BBSRC (BB/N01524X/1), Parkinson's UK (H-1202), MRC (G0801878 and M010767), and the Wellcome Trust (093445). We thank William Andrews for technical assistance.

Received: August 10, 2016
Revised: December 16, 2016
Accepted: January 19, 2017
Published: February 14, 2017

REFERENCES

Alpy, F., Rousseau, A., Schwab, Y., Legueux, F., Stoll, I., Wendling, C., Spiegelhalter, C., Kessler, P., Mathelin, C., Rio, M.C., et al. (2013). STARD3 or STARD3NL and VAP form a novel molecular tether between late endosomes and the ER. J. Cell Sci. *126*, 5500–5512.

Berridge, M.J., Bootman, M.D., and Roderick, H.L. (2003). Calcium signalling: dynamics, homeostasis and remodelling. Nat. Rev. Mol. Cell Biol. *4*, 517–529.

Brailoiu, E., Churamani, D., Cai, X., Schrlau, M.G., Brailoiu, G.C., Gao, X., Hooper, R., Boulware, M.J., Dun, N.J., Marchant, J.S., and Patel, S. (2009). Essential requirement for two-pore channel 1 in NAADP-mediated calcium signaling. J. Cell Biol. *186*, 201–209.

Burgoyne, T., Patel, S., and Eden, E.R. (2015). Calcium signaling at ER membrane contact sites. Biochim. Biophys. Acta *1853*, 2012–2017.

Calcraft, P.J., Ruas, M., Pan, Z., Cheng, X., Arredouani, A., Hao, X., Tang, J., Rietdorf, K., Teboul, L., Chuang, K.T., et al. (2009). NAADP mobilizes calcium from acidic organelles through two-pore channels. Nature *459*, 596–600.

Churchill, G.C., Okada, Y., Thomas, J.M., Genazzani, A.A., Patel, S., and Galione, A. (2002). NAADP mobilizes Ca^{2+} from reserve granules, lysosome-related organelles, in sea urchin eggs. Cell *111*, 703–708.

Davidson, S.M., Foote, K., Kunuthur, S., Gosain, R., Tan, N., Tyser, R., Zhao, Y.J., Graeff, R., Ganesan, A., Duchen, M.R., et al. (2015). Inhibition of NAADP signalling on reperfusion protects the heart by preventing lethal calcium oscillations via two-pore channel 1 and opening of the mitochondrial permeability transition pore. Cardiovasc. Res. *108*, 357–366.

Dong, R., Saheki, Y., Swarup, S., Lucast, L., Harper, J.W., and De Camilli, P. (2016). Endosome-ER contacts control actin nucleation and retromer function through VAP-dependent regulation of PI4P. Cell *166*, 408–423.

Eden, E.R. (2016). The formation and function of ER-endosome membrane contact sites. Biochim. Biophys. Acta *1861* (*8 Pt B*), 874–879.

Eden, E.R., White, I.J., Tsapara, A., and Futter, C.E. (2010). Membrane contacts between endosomes and ER provide sites for PTP1B-epidermal growth factor receptor interaction. Nat. Cell Biol. *12*, 267–272.

Eden, E.R., Sanchez-Heras, E., Tsapara, A., Sobota, A., Levine, T.P., and Futter, C.E. (2016). Annexin A1 tethers membrane contact sites that mediate ER to endosome cholesterol transport. Dev. Cell *37*, 473–483.

Favia, A., Desideri, M., Gambara, G., D'Alessio, A., Ruas, M., Esposito, B., Del Bufalo, D., Parrington, J., Ziparo, E., Palombi, F., et al. (2014). VEGF-induced neoangiogenesis is mediated by NAADP and two-pore channel-2-dependent Ca^{2+} signaling. Proc. Natl. Acad. Sci. USA *111*, E4706–E4715.

Friedman, J.R., Dibenedetto, J.R., West, M., Rowland, A.A., and Voeltz, G.K. (2013). Endoplasmic reticulum-endosome contact increases as endosomes traffic and mature. Mol. Biol. Cell *24*, 1030–1040.

Galione, A. (2015). A primer of NAADP-mediated Ca^{2+} signalling: from sea urchin eggs to mammalian cells. Cell Calcium *58*, 27–47.

Giordano, F., Saheki, Y., Idevall-Hagren, O., Colombo, S.F., Pirruccello, M., Milosevic, I., Gracheva, E.O., Bagriantsev, S.N., Borgese, N., and De Camilli, P. (2013). PI(4,5)P(2)-dependent and Ca^{2+}-regulated ER-PM interactions mediated by the extended synaptotagmins. Cell *153*, 1494–1509.

Grimm, C., Holdt, L.M., Chen, C.C., Hassan, S., Müller, C., Jörs, S., Cuny, H., Kissing, S., Schröder, B., Butz, E., et al. (2014). High susceptibility to fatty liver disease in two-pore channel 2-deficient mice. Nat. Commun. *5*, 4699.

Helle, S.C., Kanfer, G., Kolar, K., Lang, A., Michel, A.H., and Kornmann, B. (2013). Organization and function of membrane contact sites. Biochim. Biophys. Acta *1833*, 2526–2541.

Hockey, L.N., Kilpatrick, B.S., Eden, E.R., Lin-Moshier, Y., Brailoiu, G.C., Brailoiu, E., Futter, C.E., Schapira, A.H., Marchant, J.S., and Patel, S. (2015). Dysregulation of lysosomal morphology by pathogenic LRRK2 is corrected by TPC2 inhibition. J. Cell Sci. *128*, 232–238.

Idevall-Hagren, O., Lü, A., Xie, B., and De Camilli, P. (2015). Triggered Ca^{2+} influx is required for extended synaptotagmin 1-induced ER-plasma membrane tethering. EMBO J. *34*, 2291–2305.

Jongsma, M.L., Berlin, I., Wijdeven, R.H., Janssen, L., Janssen, G.M., Garstka, M.A., Janssen, H., Mensink, M., van Veelen, P.A., Spaapen, R.M., and Neefjes, J. (2016). An ER-associated pathway defines endosomal architecture for controlled cargo transport. Cell *166*, 152–166.

Kilpatrick, B.S., Eden, E.R., Schapira, A.H., Futter, C.E., and Patel, S. (2013). Direct mobilisation of lysosomal Ca^{2+} triggers complex Ca^{2+} signals. J. Cell Sci. *126*, 60–66.

Kilpatrick, B.S., Yates, E., Grimm, C., Schapira, A.H., and Patel, S. (2016). Endo-lysosomal TRP mucolipin-1 channels trigger global ER Ca^{2+} release and Ca^{2+} influx. J. Cell Sci. *129*, 3859–3867.

Levine, T.P., and Patel, S. (2016). Signalling at membrane contact sites: two membranes come together to handle second messengers. Curr. Opin. Cell Biol. *39*, 77–83.

Lin-Moshier, Y., Keebler, M.V., Hooper, R., Boulware, M.J., Liu, X., Churamani, D., Abood, M.E., Walseth, T.F., Brailoiu, E., Patel, S., and Marchant, J.S. (2014). The two-pore channel (TPC) interactome unmasks isoform-specific roles for TPCs in endolysosomal morphology and cell pigmentation. Proc. Natl. Acad. Sci. USA *111*, 13087–13092.

Luzio, J.P., Pryor, P.R., and Bright, N.A. (2007). Lysosomes: fusion and function. Nat. Rev. Mol. Cell Biol. *8*, 622–632.

Marchant, J.S., and Patel, S. (2015). Two-pore channels at the intersection of endolysosomal membrane traffic. Biochem. Soc. Trans. *43*, 434–441.

Melchionda, M., Pittman, J.K., Mayor, R., and Patel, S. (2016). Ca^{2+}/H^{+} exchange by acidic organelles regulates cell migration in vivo. J. Cell Biol. *212*, 803–813.

Naylor, E., Arredouani, A., Vasudevan, S.R., Lewis, A.M., Parkesh, R., Mizote, A., Rosen, D., Thomas, J.M., Izumi, M., Ganesan, A., et al. (2009). Identification of a chemical probe for NAADP by virtual screening. Nat. Chem. Biol. *5*, 220–226.

Pandey, V., Chuang, C.C., Lewis, A.M., Aley, P.K., Brailoiu, E., Dun, N.J., Churchill, G.C., and Patel, S. (2009). Recruitment of NAADP-sensitive acidic Ca^{2+} stores by glutamate. Biochem. J. *422*, 503–512.

Patel, S. (2015). Function and dysfunction of two-pore channels. Sci. Signal. *8*, re7.

Patel, S., and Muallem, S. (2011). Acidic Ca^{2+} stores come to the fore. Cell Calcium *50*, 109–112.

Phillips, M.J., and Voeltz, G.K. (2016). Structure and function of ER membrane contact sites with other organelles. Nat. Rev. Mol. Cell Biol. *17*, 69–82.

Pryor, P.R., Mullock, B.M., Bright, N.A., Gray, S.R., and Luzio, J.P. (2000). The role of intraorganellar Ca^{2+} in late endosome-lysosome heterotypic fusion and in the reformation of lysosomes from hybrid organelles. J. Cell Biol. *149*, 1053–1062.

Rahman, T., Cai, X., Brailoiu, G.C., Abood, M.E., Brailoiu, E., and Patel, S. (2014). Two-pore channels provide insight into the evolution of voltage-gated Ca^{2+} and Na^{+} channels. Sci. Signal. *7*, ra109.

Raiborg, C., Wenzel, E.M., Pedersen, N.M., Olsvik, H., Schink, K.O., Schultz, S.W., Vietri, M., Nisi, V., Bucci, C., Brech, A., et al. (2015a). Repeated ER-endosome contacts promote endosome translocation and neurite outgrowth. Nature *520*, 234–238.

Raiborg, C., Wenzel, E.M., and Stenmark, H. (2015b). ER-endosome contact sites: molecular compositions and functions. EMBO J. *34*, 1848–1858.

Rocha, N., Kuijl, C., van der Kant, R., Janssen, L., Houben, D., Janssen, H., Zwart, W., and Neefjes, J. (2009). Cholesterol sensor ORP1L contacts the ER protein VAP to control Rab7-RILP-p150 Glued and late endosome positioning. J. Cell Biol. *185*, 1209–1225.

Rowland, A.A., Chitwood, P.J., Phillips, M.J., and Voeltz, G.K. (2014). ER contact sites define the position and timing of endosome fission. Cell *159*, 1027–1041.

Ruas, M., Rietdorf, K., Arredouani, A., Davis, L.C., Lloyd-Evans, E., Koegel, H., Funnell, T.M., Morgan, A.J., Ward, J.A., Watanabe, K., et al. (2010). Purified TPC isoforms form NAADP receptors with distinct roles for Ca^{2+} signaling and endolysosomal trafficking. Curr. Biol. *20*, 703–709.

Ruas, M., Chuang, K.T., Davis, L.C., Al-Douri, A., Tynan, P.W., Tunn, R., Teboul, L., Galione, A., and Parrington, J. (2014). TPC1 has two variant isoforms, and their removal has different effects on endo-lysosomal functions compared to loss of TPC2. Mol. Cell. Biol. *34*, 3981–3992.

Sakurai, Y., Kolokoltsov, A.A., Chen, C.C., Tidwell, M.W., Bauta, W.E., Klugbauer, N., Grimm, C., Wahl-Schott, C., Biel, M., and Davey, R.A. (2015). Ebola virus. Two-pore channels control Ebola virus host cell entry and are drug targets for disease treatment. Science *347*, 995–998.

Stern, M.D. (1992). Buffering of calcium in the vicinity of a channel pore. Cell Calcium *13*, 183–192.

Stuible, M., Abella, J.V., Feldhammer, M., Nossov, M., Sangwan, V., Blagoev, B., Park, M., and Tremblay, M.L. (2010). PTP1B targets the endosomal sorting machinery: dephosphorylation of regulatory sites on the endosomal sorting complex required for transport component STAM2. J. Biol. Chem. *285*, 23899–23907.

Yamasaki, M., Thomas, J.M., Churchill, G.C., Garnham, C., Lewis, A.M., Cancela, J.M., Patel, S., and Galione, A. (2005). Role of NAADP and cADPR in the induction and maintenance of agonist-evoked Ca^{2+} spiking in mouse pancreatic acinar cells. Curr. Biol. *15*, 874–878.

Cell Reports, Volume *18*

Supplemental Information

An Endosomal NAADP-Sensitive Two-Pore Ca^{2+} Channel Regulates ER-Endosome Membrane Contact Sites to Control Growth Factor Signaling

Bethan S. Kilpatrick, Emily R. Eden, Leanne N. Hockey, Elizabeth Yates, Clare E. Futter, and Sandip Patel

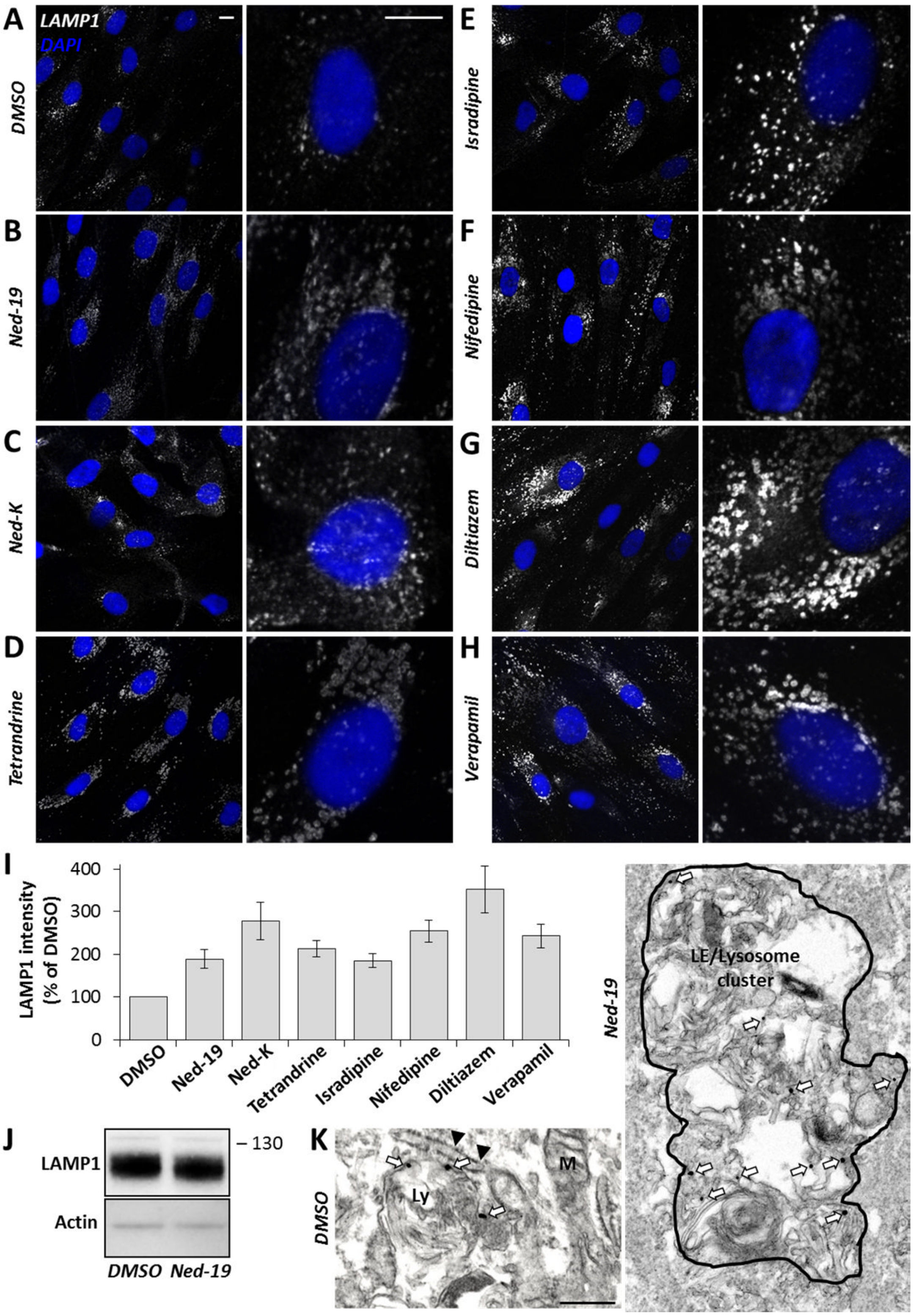

Figure S1. TPCs maintain late endosome/lysosome morphology, Related to Figure 1.

(A-H) Representative confocal images of LAMP1 staining (white) in fibroblasts treated with either DMSO (0.1%; A), the NAADP antagonists Ned-19 (100 µM, B) and Ned-K (100 µM, C), or the Ca^{2+} channel blockers tetrandrine (10 µM, D), isradipine (100 µM, E), nifedipine (100 µM, F), diltiazem (100 µM, G) and verapamil (100 µM, H) for 2h. Nuclei were stained using DAPI (blue). Zoomed images are displayed in the right panels. Scale bars, 10 µm.

(I) Summary data quantifying LAMP1 intensity as a percentage of DMSO control. Data from 39-49 cells from 3 independent platings.

(J) Representative Western blot using antibodies to LAMP1 (top) or actin (bottom) and homogenates from fibroblasts treated with Ned-19 (100 µM) overnight.

(K) Representative electron micrographs of LAMP1 (white arrows) labelling in fibroblasts treated with either DMSO (0.1%) or Ned-19 (100 µM). Typical cluster of LAMP1-positive late endosome/lysosomes is outlined in black. Ly, lysosome; M, mitochondria; black arrowheads, ER. Scale bar, 300nm.

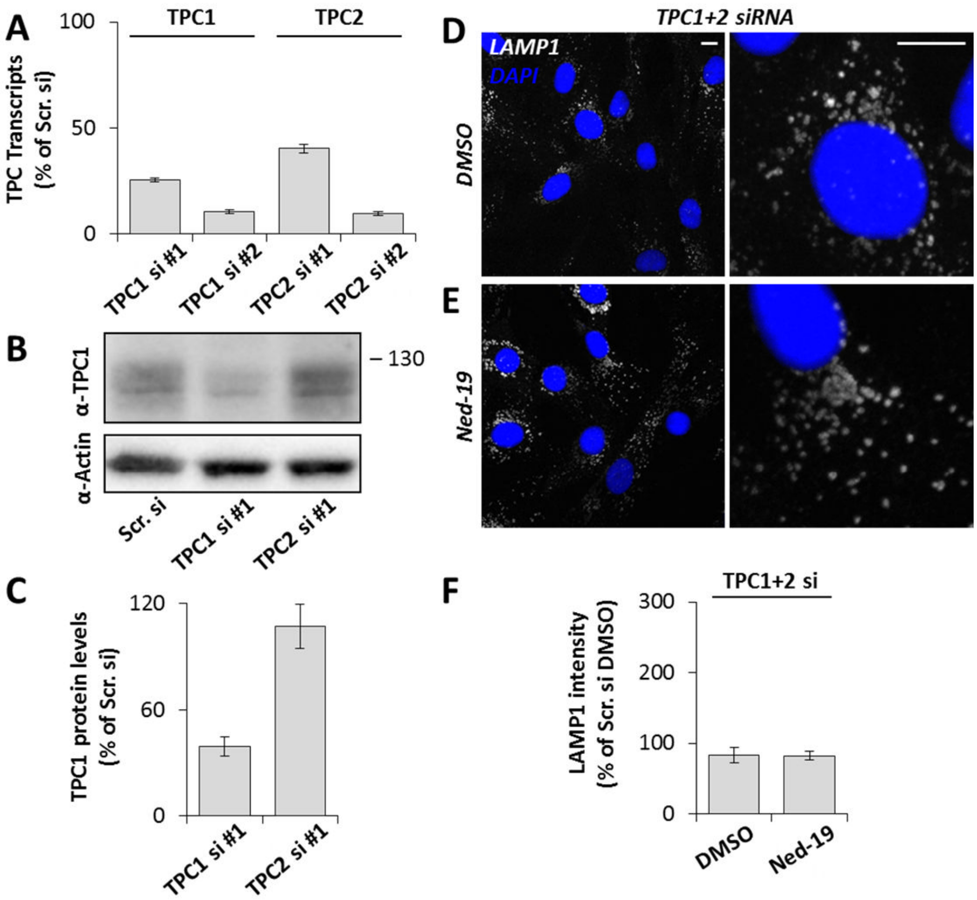

Figure S2. Knockdown of TPCs, Related to Figure 2.

(A) Quantitative PCR analysis of TPC1 (left) and TPC2 (right) levels in fibroblasts transfected with the indicated TPC siRNA for 48h. Data are from 1-4 experiments (analysing 1-2 fibroblast lines) and normalised to TPC levels in cells transfected with scrambled (Scr) siRNA.

(B) Representative Western blot using antibodies to TPC1 (top) or actin (bottom) and homogenates from fibroblasts treated with the indicated siRNA.

(C) Summary data quantifying TPC1 protein levels from 6 independent knockdowns. Data are normalised to TPC1 levels in cells treated with scrambled siRNA.

(D-E) Representative LAMP1 staining (white) in fibroblasts co-transfected with siRNAs targeting TPC1 and TPC2 for 48h and treated for the last 18h with either DMSO (0.1%, D) or Ned-19 (100 µM, E). Nuclei were stained using DAPI (blue). Zoomed images are displayed in the right panels.

(F) Summary data quantifying LAMP1 intensity as a percentage of DMSO. Data are from 39-40 cells from 3 independent knock-downs.

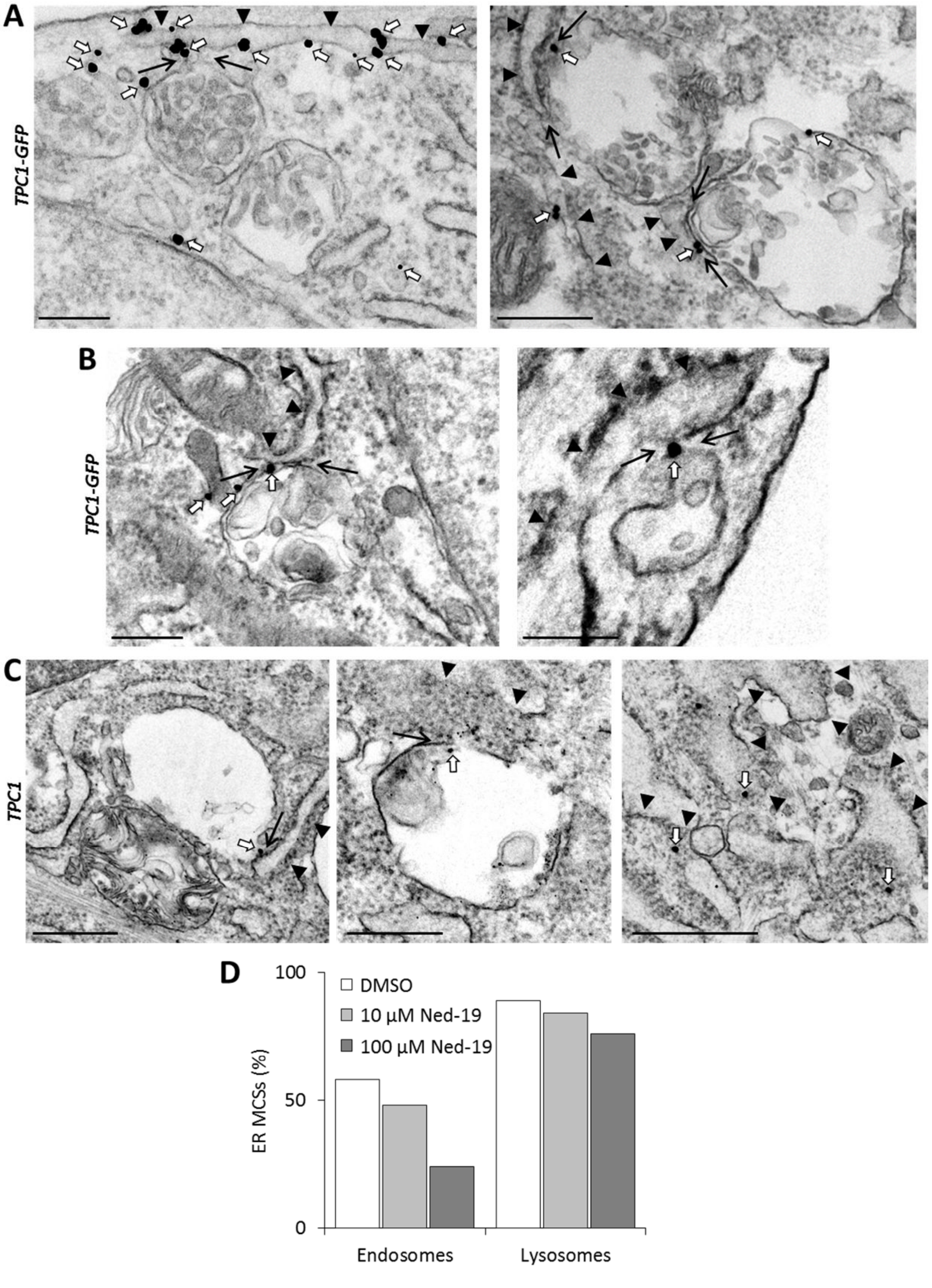

Figure S3. TPC1 localises to ER-endosome contact sites and regulates their formation, Related to Figure 4.

(A-C) Electron micrographs showing distribution of TPC1 in fibroblasts. Cells were transfected with TPC1-GFP (A-B) or untagged TPC1 (C) and stained for GFP/TPC1 using pre-embedding labelling. TPC1 (white arrows) partially localises to the ER (arrowheads) as well as endosomes and to ER-endosome membrane contact sites (black arrows). Scale bar, 200 nm.

(D) Analysis of ER-endosome or lysosome membrane contact sites in Hela cells treated with DMSO (0.1%) or Ned-19 (10 or 100 µM) overnight.

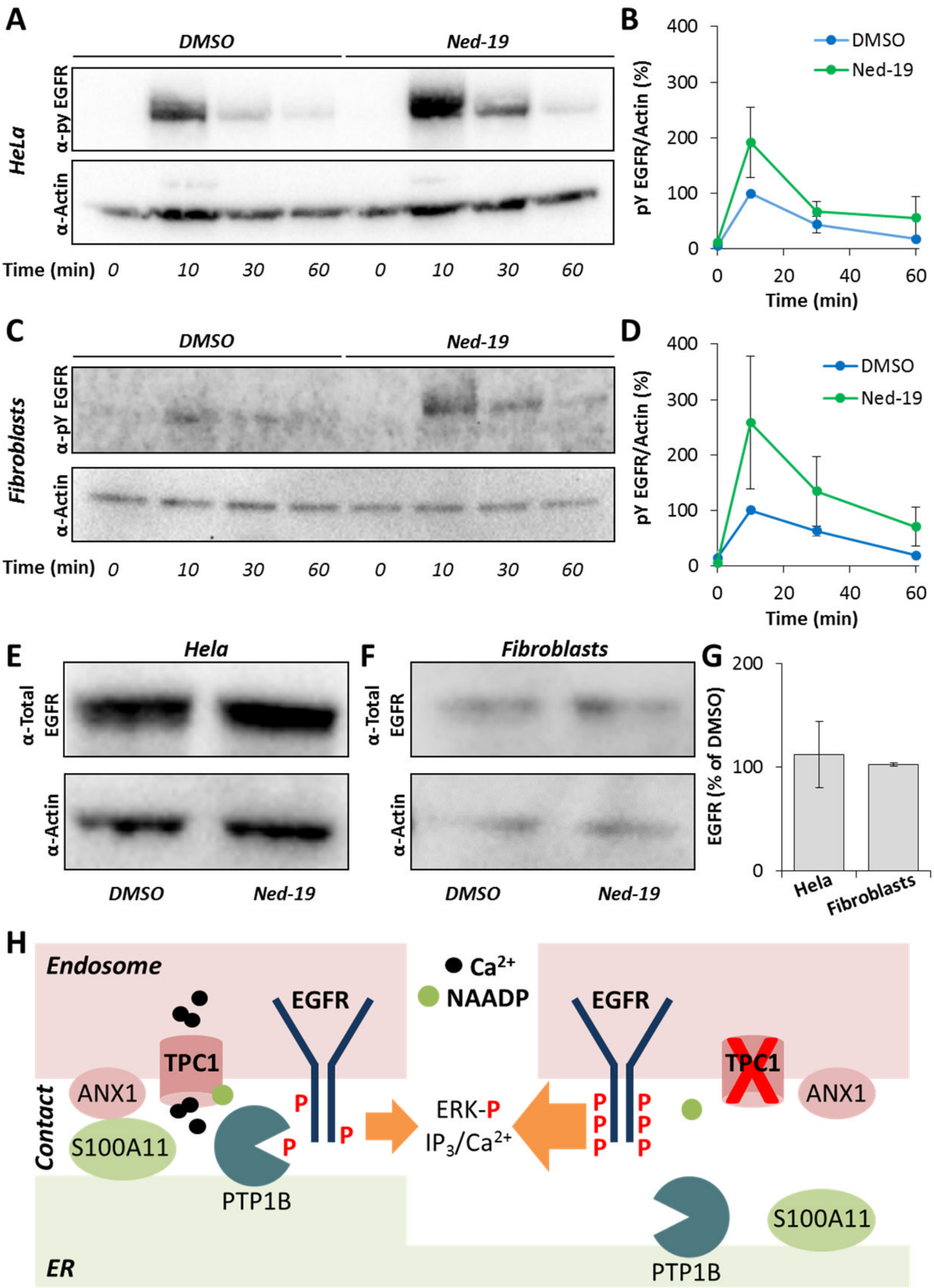

Figure S4. NAADP regulates EGF signalling, Related to Figure 5.

(A-D) Western blot using antibodies to phosphotyrosine (pY) 1068 EGFR (top) or actin (bottom) and homogenates from Hela cells (A) or fibroblasts (C) treated with either DMSO (0.1%) or Ned-19 (100 µM) overnight and serum starved for 1h prior to EGF (100 ng/mL) stimulation at the indicated times. Summary data analysing phosphotyrosine-EGFR receptor levels (normalised to actin) in lysates from Hela cells (B) or fibroblasts (D) quantified as a percentage of DMSO control 10 minutes after EGF stimulation. Data are from 3 independent treatments.

(E-F) Western blot using antibodies to total EGFR (top) or actin (bottom) and homogenates from Hela cells (E) or fibroblast (F) treated with either DMSO (0.1%) or Ned-19 (100 µM) overnight.

(G) Summary data analysing total EGFR receptor levels in lysates quantified as a percentage of DMSO control. Data are from 3-4 independent treatments.

(H) Left, schematic depicting local NAADP-dependent Ca^{2+} flux through TPC1 at membrane contact sites between the endosomes and ER. These sites are populated by ANX1/S100A11 and the ER-localised protein phosphatase PTP1B which dephosphorylates endocytosed EGFRs during stimulation thereby limiting signalling through downstream pathways such as ERK and PLCγ (small arrow). Right, disrupting NAADP/TPC1 signalling (red cross) reduces contact resulting in greater phosphorylation of EGF receptors and enhanced signalling (large arrow).

Treatment	Time	Endo-lysosome size (μm^2)	n
DMSO (0.1 % v/v)	Overnight	0.42 ± 0.005	5256
Ned-19 (100 µM)	Overnight	0.47 ± 0.007	4222
Ned-K (100 µM)	Overnight	0.53 ± 0.009	6734
Tetrandrine (10 µM)	Overnight	0.55 ± 0.08	3892
DMSO (0.1 % v/v)	2h	0.36 ± 0.005	4379
Ned-19 (100 µM)	2h	0.42 ± 0.005	8415
Ned-K (100 µM)	2h	0.45 ± 0.006	6734
Tetrandrine (10 µM)	2h	0.65 ± 0.01	3892
Isradipine (100 µM)	2h	0.45 ± 0.001	7489
Nifedipine (100 µM)	2h	0.47 ± 0.006	6796
Diltiazem (100 µM)	2h	0.51 ± 0.008	6883
Verapamil (100 µM)	2h	0.52 ± 0.007	5537
Control siRNA	72h	0.40 ± 0.007	14600
TPC1 siRNA	72h	0.43 ± 0.005	29199
TPC2 siRNA	72h	0.39 ± 0.004	19663

Table S1. NAADP/TPC signalling maintains late endosome/lysosomal morphology, Related to Figures 1 and 2.

Effect of the indicated drug or siRNA treatment for the given time on late endosome/lysosome size. The area of the indicated number (n) of LAMP1-labelled structures were quantified from confocal micrographs from 3 independent experiments.
